# History of SCS and patient-focused innovations: a manufacturer’s perspective on the role of technology

**DOI:** 10.1093/pm/pnag040

**Published:** 2026-06-10

**Authors:** Michael A Fishman, John A Hatheway, Krishnan Chakravarthy, Jan Willem Kallewaard, Alaa Abd-Elsayed, Leonardo Kapural, Sean Li, Yashar Eshraghi, Melissa Murphy, Navdeep Jassal, Peter Staats, Jacob Caylor, Prabhdeep K Grewal, Lisa M Johanek, Julia E Gamache

**Affiliations:** Michael Fishman PLLC, Lebanon, PA, 17042, United States; Brixton Biosciences, Cambridge, MA, 02138, United States; Columbia Pain Management, Hood River, OR, 97031, United States; VA San Diego Healthcare, San Diego, CA, 92161, United States; Innovative Pain Treatment Solutions and Surgery Centers, San Diego, CA 92111, United States; Solaris Research Institute, San Diego, CA, USA; Department of Anesthesiology and Pain Management, Rijnstate Hospital, Arnhem, 6815 AD, Netherlands; Department of Anesthesiology and Pain Management, Amsterdam University Medical Center, Amsterdam, 1105 AZ, Netherlands; Department of Anesthesia, University of Wisconsin, Madison, WI, 53715, United States; Carolinas Pain Institute and Queen City Clinical Research, Winston-Salem, NC, 27103, United States; Premier Pain Centers, Shrewsbury, NJ, 07702, United States; Ochsner Health System, New Orleans, LA, 70115, United States; North Texas Orthopedics & Spine Center, Grapevine, TX, 76051, United States; EXCEL Pain and Spine, Brandon, FL, 33511, United States; National Spine and Pain Centers, FL, 32233, United States; Central Texas Pain Center PLLC, Cedar Park, TX, 78613, United States; Pain Management, Burkhart Research Institute for Orthopaedics, San Antonio, TX, 78216, United States; Medtronic Neuromodulation, Minneapolis, MN, 55432, United States; Medtronic Neuromodulation, Minneapolis, MN, 55432, United States

**Keywords:** spinal cord stimulation, technology, chronic pain, closed-loop, neurostimulator

## Abstract

**Background:**

The first case study of spinal cord stimulation (SCS) for pain treatment, published in 1967, catalyzed decades of research to improve and optimize this therapy.

**Content Overview:**

This article provides an overview of the history of SCS technology, including advances made by industrial and academic innovators in lead designs, neurostimulator features, and battery chemistry. Foundational randomized controlled trials (RCTs) and a timeline of key product releases from the early 1970s are highlighted. The second half of the article discusses SCS technology advancements from a manufacturer’s perspective and how they relate to improving the overall patient therapy experience.

**Conclusions:**

Specific SCS technology advancements that have focused on the patient’s needs include: The development of patient programmers, enhanced rechargeable battery technology, position-adaptive neurostimulators, full-body magnetic resonance imaging (MRI) conditionality, and sensing-based closed-loop features.

## SCS system evolution

In 1965, Melzack and Wall proposed the famous gate control theory of pain perception, in which pain could be “blocked” by closing or occupying the “gate” with non-painful stimuli.^1^ This theory sparked experimentation with electrical stimulation to target human peripheral nerves and the feline dorsal columns, demonstrating the ability to block painful sensations.^2^ Thomas Mortimer, a graduate student at the Case Institute of Technology, designed the first crude spinal cord stimulation (SCS) device in collaboration with Dr. Norm Shealy. Utilizing a circuit diagram of a radiofrequency receiver provided by a colleague at Medtronic, Mortimer constructed an implantable radiofrequency dorsal column stimulator–only two such devices were ever built. In 1967, Shealy implanted one of these devices into a 70-year-old man.^3^ The patient’s response to stimulation was carefully monitored over 1.5 days with encouraging results, including a dramatic elimination of his pain.^4^ This groundbreaking work was featured in the first-ever case study of SCS for pain management and marked the beginning of foundational clinical publications supporting SCS (Table 1). The development of SCS systems occurred at a time when cardiac pacemakers had already achieved early clinical success. By the early 1960s, pacemakers were fully implantable electronic devices with long-lived power sources, transitioning from mercury-zinc to more durable lithium-iodide cells.^22^ These advances, along with hermetically sealed titanium housings, corrosion-resistant conductors, and improved electrode materials, offered engineering solutions applicable to chronic neurostimulation.^22^ Over time, concepts from pacing, such as sensor-based features and MRI conditionality, continued to inform SCS system design.^23^ Nevertheless, differences in therapeutic requirements let to important divergences, such as the need to stimulate a mobile neural target and the wide patient variability in pain location, pain conditions, and neuroanatomy. While a detailed comparison is beyond the scope of this manuscript, appreciating SCS development in the context of cardiac device development provides valuable context for the evolution of today’s SCS systems.

**Table 1:** Timeline of key SCS studies and product releases.

Year	Brief description
**1967**	First SCS case report (Shealy et al, 1967)
**1968**	Medtronic released the Myelostat RF-coupled system used for SCS
**1972**	Medtronic introduced Model 3600 acute percutaneous screening system
**1974**	Medtronic introduced the first quadripolar percutaneous lead, PISCES™, for permanent implant
**1978**	Medtronic introduced Resume™ 4-contact surgical (paddle-shaped) lead for permanent implant
**1981**	Neuromed released programmable RF system with a 4-contact lead
**1981**	Cordis released first fully implantable SCS system (system with a self-contained, battery-driven power source)
**1984**	Medtronic released the Itrel™ neurostimulator
**2000**	RCT for SCS + PT vs PT alone in patients with CRPS (Kemler et al, 2000)
**2004**	ANS (formerly Neuromed) released first anatomically contoured surgical (paddle) lead
	Boston Scientific acquired SCS technology from Advanced Bionics
	Boston Scientific released first rechargeable neurostimulator
**2005**	RCT for conventional SCS vs reoperation for patients with FBSS (North et al, 2005)
	ANS released first transverse tripolar surgical lead
	St. Jude acquired ANS
	St. Jude released first system with 4 independently programmable leads
	Boston Scientific released first 16-contact surgical lead with tight spacing
	Medtronic released their first rechargeable neurostimulator
	Medtronic released an MRI-compatible percutaneous system for head scans
**2007**	PROCESS study: SCS + CMM vs CMM alone in patients with FBSS (Kumar et al, 2007)
	Medtronic released the Specify™ 5-6-5 surgical Lead
**2009**	St. Jude released first 5-column surgical lead
	Medtronic released the Specify™ 2x8 surgical Lead
**2011**	Boston Scientific released first 16-contact percutaneous lead
**2012**	Medtronic released AdaptiveStim™ technology for position-adaptive stimulation
	RestoreSensor RCT: SCS position-adaptive stimulation vs SCS manual programming (Schultz et al, 2012)
**2013**	Boston Scientific released first neurostimulator that could accommodate 32 independent channels
	Medtronic released SureScan™ MRI lead technology for full body MRI conditionality
	Medtronic Vectris™ lead approved for full body MRI scans
**2014**	Boston Scientific released first 32-contact surgical lead
**2015**	Nevro released HF10™ therapy for SCS
**2015**	SENZA RCT: HF10™ SCS vs conventional SCS in patients with chronic back and leg pain (Kapural et al, 2015)
**2016**	Medtronic released Specify™ SureScan™ MRI 5-6-5 and 2 × 8 leads for full-body MR conditionality
**2016**	St. Jude released BurstDR™ therapy for SCS
**2017**	Abbott acquired St. Jude Medical
**2018**	SUNBURST RCT: BurstDR™ SCS vs conventional SCS (Deer et al, 2018)
**2020**	EVOKE RCT: Closed-loop SCS vs conventional SCS (Mekhail et al, 2020)
	WHISPER RCT: ≤ 1.2 kHz SCS vs conventional SCS (North et al, 2020)
**2021**	RCT for DTM™ SCS vs conventional SCS (Fishman et al, 2021)
	SENZA DPN RCT: Comparison of HF10™ SCS plus CMM to CMM for painful diabetic peripheral neuropathy (Petersen et al, 2021)
**2022**	Saluda Medical released the first closed-loop ECAPs-based SCS system
	RCT comparing HF10™ SCS plus CMM to CMM alone for nonsurgical refractory back pain (Kapural et al, 2022)
**2023**	COMBO RCT: Conventional SCS vs dual use of sub- and supra- perception SCS (Wallace et al, 2023)
	DISTINCT RCT: BurstDR™ SCS vs CMM in patients with PSPS-T1 ineligible for spinal surgery (Deer et al, 2023)
	Biotronik released the first SCS system capable of remote monitoring
**2024**	Medtronic released Inceptiv™, an SCS system with a closed-loop ECAPs-based feature
	NOVA RCT: DTM™ SCS vs conventional SCS for DDD, HD, or RPS patients with chronic back pain ineligible for spine surgery (White et al, 2024)
	EU DTM SCS RCT: DTM™ SCS vs CMM for PSPS-T1 in patients ineligible for spinal surgery (Kallewaard et al, 2024)
**2025**	SOLIS RCT: SCS vs CMM in PSPS-T1 patients (North et al, 2025)

The blue shaded rows indicate foundational studies.^4–20^ The products launched in the United States listed in the table are limited to the paradigm-shifting events discussed in this article and do not constitute an exhaustive list. A similar timeline has been published elsewhere.^21^

Abbreviations: CMM, conventional medical management; COMBO, Combining Mechanisms for Better Outcomes; CRPS, complex regional pain syndrome; DDD, degenerative disc disease; DISTINCT, Dorsal spInal cord STImulatioN vs mediCal management for the Treatment of low back pain; DTM, differential target multiplexed; ECAP, evoked compound action potential; FBSS, failed back surgery syndrome; HD, herniated disc; HF, high frequency; PROCESS, Prospective Randomized Controlled Multicenter Trial of the Effectiveness of Spinal Cord Stimulation; PSPS-T1, persistent spinal pain syndrome type 1; PT, physical therapy; RPS, radicular pain syndrome; SUNBURST, Success Using Neuromodulation With BURST; WHISPER, Effectiveness of the Precision Spinal Cord Stimulator System at Sub-Perception Amplitude.

### Leads: From plates to percutaneous and surgical leads

Fundamental publications following Shealy’s case study largely centered on lead design. Initially, SCS leads consisted of a single column of contacts (electrodes) fixed to a plate, mesh, or pronged electronic circuit board, which required surgical implantation (Figure 2A).^24^ This type of surgical placement was fraught with complications such as cerebrospinal fluid (CSF) leak, fibrosis, increasing electrode impedance, and spinal cord compression. These complications were primarily associated with the surgical implant techniques used at the time, such as subdural and endodural (intradural) placements,^25^ which are now considered off-label and have been replaced by the current on-label practice of epidural lead placement. One of the earliest systems—the Myelostat, released in 1968^26^—required a laminectomy for implantation because its plate was secured directly to the dura.^27^ In addition, the system was designed as a single integrated unit, with the cathodic plate permanently fixed to the receiver and a wired anode implanted intramuscularly. Separating the lead from the receiver led to the development of multicontact leads with both anode and cathode on a single lead, enabling minimally invasive percutaneous delivery. Percutaneous leads, which could be introduced into the epidural space through a Tuohy needle, were developed by Medtronic in the mid-1970s (Table 1).^28^^,^^29^ The use of percutaneous leads during an SCS trial was first described in 1975.^28^ Of the 26 patients receiving temporary SCS leads in this study, 15 achieved satisfactory results, and 10 received a permanent implant. Thus, the first reported permanent SCS implant-to-trial ratio was 38.5%.

The first reported case series of chronic stimulation (six months) with percutaneously inserted epidural electrodes was published in 1977 by North et al.^30^^,^^31^ The case series involved 31 patients with varying sources of intractable pain. 17 patients experienced 70%–100% pain relief, and 7 experienced 40%–70% relief. In addition, patients reported improvements in physical function and reductions in pain-related medication use. Frequent lead migration and lead fracture requiring surgical intervention were also reported. They concluded the publication by highlighting issues with system reliability and patient convenience. These publications were impactful because they provided a call to action for technological innovation of SCS systems. Furthermore, they facilitated access to the therapy by other specialties, such as anesthesiologists, by demonstrating its success with easily placed percutaneous leads.^24^

What followed was a rapid advancement in lead design, spurred primarily by three factors: (1) A desire for greater configurability, made possible by increasing the number of electrodes; (2) An understanding that contact spacing can directly influence the paresthesia threshold; and (3) The need for energy efficient lead designs to be compatible with batteries, which, at the time, had limited longevity (Table 2).^32^^,^^33^

**Table 2:** Key technological innovations for the improvement of SCS lead designs.

Initial technology	Motivator for improving SCS therapy	Innovative development
Two-contact bipole	Loss of coverage due to lead migration or development of new pain areas	Leads with 4, 8, 16, 32 contacts; changes in contact spacing and contact length
Single-channel stimulation; intraoperative pain-paresthesia mapping; in-office reprogramming; surgical lead revision	Precise targeting of paresthesia to the patient’s pain area (eg, back pain)	Computerized multi-contact programming; complex field shaping; transverse tripole; current steering; interleaving
Percutaneously placed leads	Lead migration and a need to reduce energy consumption^a^ and stabilize electrode contact configurations	Surgical (paddle) leads; tripolar paddles; advances in anchors and anchoring techniques

a As discussed, surgical leads have lower amplitude requirements and thus reduce energy consumption.

**Figure 1:**
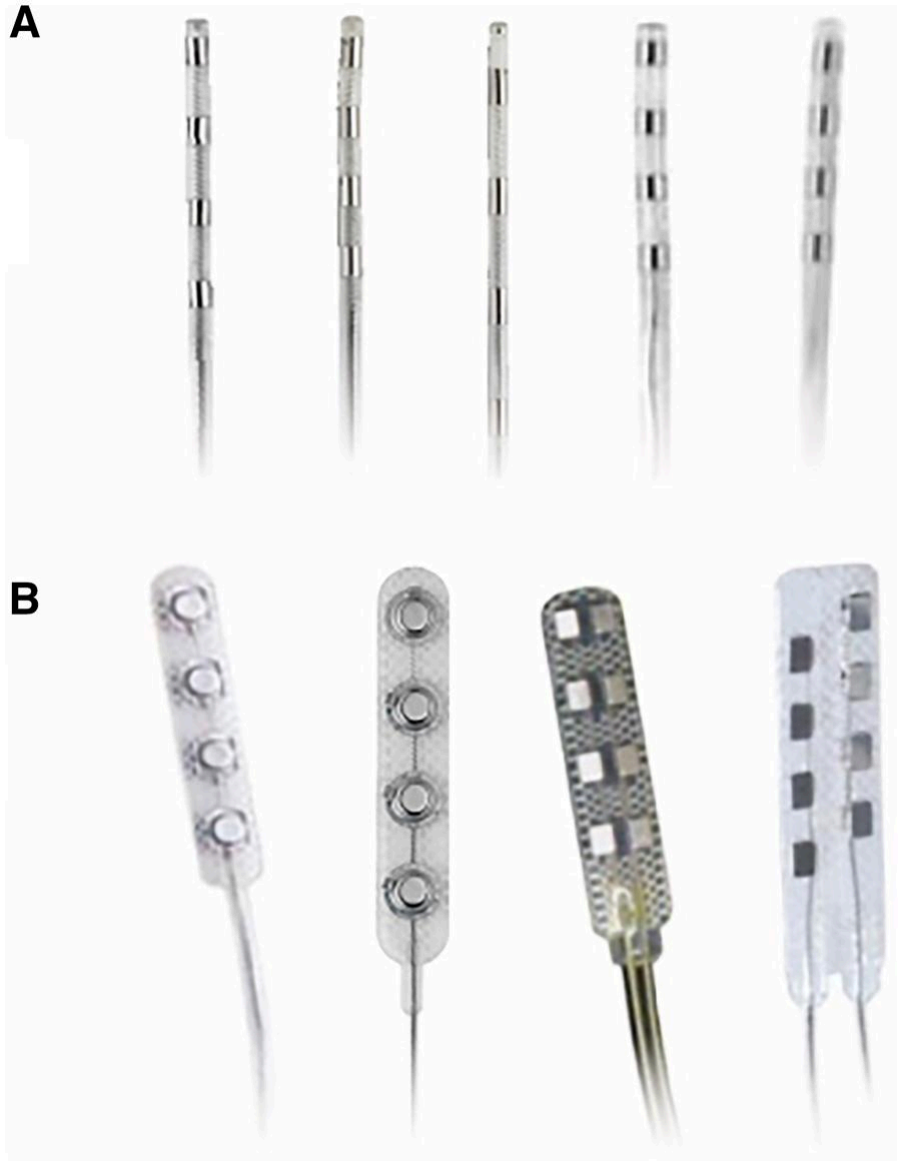
Examples of early Medtronic percutaneous and surgical SCS leads. (A) Percutaneous leads from left to right: Pisces-Quad, Pisces-Quad Compact, Pisces-Quad Plus, Pisces-Quad LZ, Pisces-Quad. (B) Surgical leads from left to right: Resume TL 1x4, Resume II 1x4, Specify 2x4, Specify 2x4 hinged. Reprinted with permission from © Medtronic, Inc.—all rights reserved.

Medtronic laid the groundwork in the 1970s by introducing a monopolar percutaneous lead composed of a stainless steel coil and a bare stimulating tip, intended for temporary stimulation during an SCS trial.^28^ Significantly, this introduction of a lead that could be implanted percutaneously facilitated the broad adoption of the therapy by nonsurgeons. Neuromed, in 1981, released a lead with an inline 4-contact array, which was novel at the time and eliminated the need for multiple single-contact leads.^24^ Furthermore, the RF system associated with this device can be programmed after implantation, enabling noninvasive adjustment of programming parameters. That same decade, Medtronic developed multi-polar leads for permanent implant (see Table 1 and Figure 1A). A unique development at the time was a 4-contact lead from Medtronic that included a temporary extension for testing contacts and parameters. The wires at the end of the extension were graded in length, indicating the corresponding contact on the lead.^34^

Technological advances in the 1980s enabled the introduction of eight-contact percutaneous leads. In parallel, a different type of lead was being developed—surgical leads, also called paddle leads or insulated leads, which could accommodate multiple (two to five) columns of electrodes and were placed via laminotomy (Figure 1B). In the early 2000s, surgical leads were argued to have several technical advantages, including achieving specific patterns of paresthesia coverage, lower amplitude requirements, and reduced migration rates.^35^^,^^36^ However, some studies have found no difference in lead migration rates between percutaneous and surgical leads.^37^^,^^38^ Many percutaneous and surgical lead design innovations continued through 2014, eventually achieving up to 32 programmable electrodes in a single SCS system (Table 1).^24^^,^^39^

The increase in electrodes per lead led to more potential programming configurations, allowing for more precise paresthesia coverage. During this time, pivotal publications focused on optimal electrode spacing, as researchers such as Law^40^ and Holsheimer^41^ showed that closely spaced electrodes were most effective for dorsal column fiber recruitment and driving paresthesia to the lower back. Configurations that selectively target the dorsal columns were shown to reduce lateral stimulation near the dorsal roots, which can contribute to discomfort.^42^

During this time, investigators also began reporting the need for optimal paresthesia coverage for complex back and leg pain^33^^,^^43^^,^^44^ as it became clear that changing the position of the positively (anode) and negatively (cathode) charged contacts influenced the distribution of paresthesia. Specifically, increasing the distance between the anode and cathode increased the range of activated structures at the expense of resolution. Thus, contact length was found to affect impedance, while the distance between contacts affected resolution; this knowledge informed optimal lead design and stimulation parameters.^41^ Some clinicians used one percutaneous quadripolar lead placed on the physiologic midline, with the cathode placed at T8 or T9 and the anode above or below it, respectively.^40^^,^^45^ Others experimented with various other side-by-side percutaneous lead configurations, such as a “transverse tripole” (a central cathode with two flanking anodes),^42^ to achieve effective paresthesia for low back pain (Table 2). Practical shaping of the electrical field to overlap painful areas is best achieved when the leads are initially placed at an optimal position.^46^ Several innovations have been introduced over the years to enhance the steerability of percutaneous leads during placement, including improved lead flexibility and a variety of stylets.^47^

### Neurostimulators: From radiofrequency systems to rechargeables

Today, neurostimulator devices are available with three main power technologies: Wireless coupling (microwave or RF), primary cell, and rechargeable cells.^48^ In the following section, we review these three power technologies in the order of their historical development: Wireless coupling (RF-powered devices), primary cell neurostimulators, and rechargeable systems. Regardless of the technology used to power the system, SCS requires a large power source for adequate functionality, so the power source and battery technology are ongoing areas of advancement.

**Figure 2:**
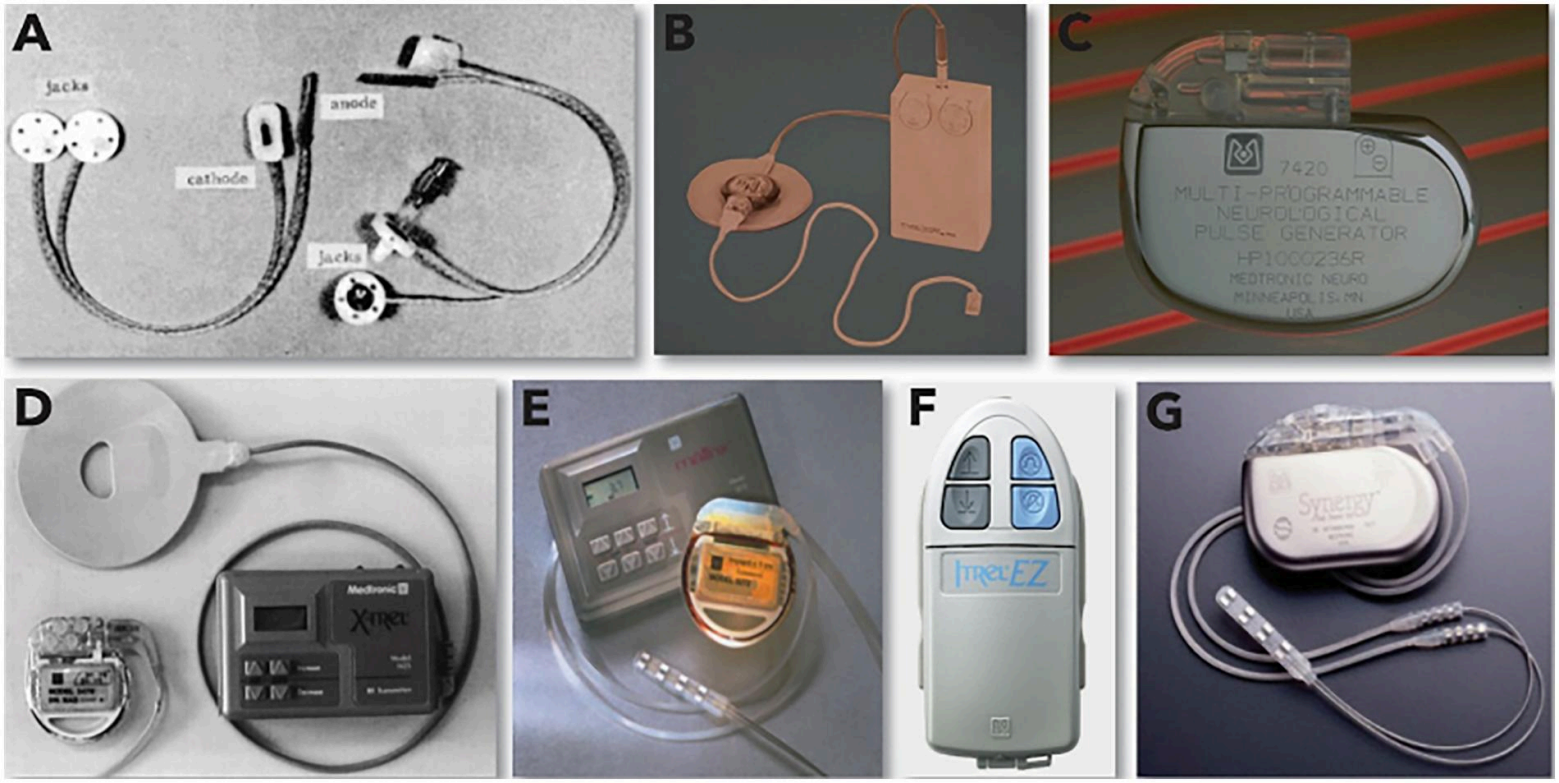
SCS devices were available from 1967 to 1999. (A) 1967—The first spinal cord stimulator. (B) 1968—The Myelostat RF-coupled system. (C) 1982—Itrel, the first fully implantable SCS neurostimulator. (D) 1988—The Xtrel RF-coupled system supported 4 electrodes, 2 on each lead. (E) 1995—The Mattrix RF-coupled system, which supported 8 electrodes. (F) 1995—The EZ Patient Programmer, compatible with the Itrel 3 system. (G) 1999—The Synergy system, which supported 8 electrodes and had expanded programming options. Reprinted with permission from © Medtronic, Inc. —all rights reserved.

Until the late 1970s, SCS systems were either externally powered radiofrequency (RF) systems (Figure 2E) or implantable stimulators that could only be reprogrammed with invasive device access.^24^ RF systems used RF-coupled transmission and electromagnetic induction to transmit power from an external power source worn by the patient (Figure 2B). Importantly, these systems had the novel advantage of allowing for noninvasive adjustment of programming parameters and a relatively broad range of programming options, since there were no long-term energy constraints (although some systems used disposable batteries).^49^

However, there were several issues associated with this technology, including a requirement for additional electrode interventions;^50^ the need for higher amplitudes or frequent parameter adjustments to sustain clinical benefit; ^51^ the potential for contact dermatitis and swelling caused by materials needed to maintain proper position of the transmitting coil over the receiving coil; ^51^ and significant limitation of patient daily activities due to the bulky and cumbersome build of these devices.^24^^,^^48^

Discoveries in battery chemistry, specifically the development of lithium batteries, prompted research into fully implantable neurostimulators with on-board controllers and batteries.^49^ This allowed Cordis Inc., a Miami-based pacemaker company, to introduce the Omnicor lithium-powered SCS stimulator in 1980. The first clinical trial of this type of device began in 1982 with Medtronic’s Itrel neurostimulator and led to FDA approval in 1984 (Table 1, Figure 2C).^52^ Internally powered SCS systems gained more widespread use in the following decades. Primary cell neurostimulators offer several benefits—the entire circuit is implanted in the patient and can be used during normal day-to-day activities. Because primary cell devices do not require regular recharging, patients are not burdened with the need to remember frequent charging sessions. This simplicity not only enhances ease of use but also promotes consistent therapy delivery, as there is no risk of therapy interruption due to a missed charging session.

However, primary cell neurostimulators have several drawbacks. First, reliable therapy delivery has a finite period, typically less than 10 years, due to the limited battery capacity. Compared to cardiac pacemakers, the operational lifetime of these stimulators is shorter, as spinal cord stimulation requires substantially more energy—both because SCS devices are typically in continuous use and because they require greater current to activate neural tissue. With manual programming by the physician, initial estimates of maximum battery life of Itrel 3 and Synergy (Medtronic) neurostimulators averaged 3.8 years (Figure 2G); however, settings can be further optimized for longevity as demonstrated by the use of an investigational patient programmer that increased battery life to 4.6 years in a research study.^53^ Current iterations may have an average lifespan of up to 8.5 years; however, this can be significantly lower depending on the manufacturer and stimulation parameters.^54^^,^^55^ Second, the need for more frequent device replacements leads to additional surgical procedures and a greater risk of complications, which may interrupt a therapy that is providing effective pain relief.^56^ Each additional SCS surgical procedure carries risks of complications, such as infection, to which the patient is again exposed.^57^ This anticipated need for replacement may also discourage some patients from selecting primary cell systems, especially if they consider the aforementioned challenges associated with undergoing additional procedures. Finally, with a primary cell, the range of programming options is limited to those with low energy consumption to prevent battery drain.

In 2004, the advent of a rechargeable cell SCS neurostimulator by Boston Scientific led to significant improvements in programming flexibility and the range of activities patients could continue to perform while undergoing treatment (Table 1). Based on bench testing, the first FDA-approved rechargeable battery’s life was calculated at 10–25 years.^56^ However, clinical data show that the median time to explant for rechargeable devices is 7.2–8.2 years.^58^^,^^59^ Neurostimulator replacement needs are also reduced with rechargeable systems, requiring 2 to 4 fewer replacements due to battery depletion than primary cell systems.^56^ These SCS systems require regular recharging, which may be scheduled from daily to every few weeks, with charging sessions ranging from 30 minutes to 6 hours.^60^ The frequency of the recharge interval depends on various factors. For instance, patients can choose from different stimulation modes, from continuous to various cycling methods, which affect recharge intervals differently.

Additionally, larger batteries, including those with longer interval times, require longer recharge times when depleted. Lithium-ion cells in rechargeable neurostimulators each have a finite number of charge-discharge cycles before gradual degradation. Thus, the battery loses capacity over time, altering the recharge interval.^48^ Lithium-ion cells with improved long-term battery capacity have been a recent area of technology innovation.

The evolution of SCS power technologies reflects ongoing innovation aimed at improving patient convenience, device longevity, and therapeutic flexibility. Continued advancements in battery chemistry and power management are expected to provide patients with additional options and further enhance the usability of SCS systems.

## Focusing on patient needs and user experience

Many design advancements mentioned above were developed in response to specific patient needs. As SCS therapy has matured, the neuromodulation industry has maintained a steadfast commitment to enhancing the patient experience throughout their journey with SCS, recognizing that the patient experience extends far beyond the specific therapy employed (Box 1). SCS device manufacturers have increasingly focused on improving the patient experience, as evidenced by the product launches and clinical studies in Table 2. While a comprehensive industry-wide assessment is beyond the scope of this review, the following section highlights several examples from a single device manufacturer that illustrate a patient-centric approach. These include developments in battery technology, position-adaptive and closed-loop stimulation, and full-body MR Conditional devices.

### Early entry into the fully implantable neurostimulator

A lithium-thionyl chloride battery was developed and incorporated into neurostimulators to manage the high rates and amplitudes required for neurostimulation (versus cardiac pacing).^49^ As mentioned above, the fully implantable neurostimulator–defined as having a self-contained, battery-driven power source—became commercially available in 1984 (Itrel; Table 1). Compared with prior RF systems powered by an external power source (Figure 2E), an implantable neurostimulator offered many advantages for patients, including continuous stimulation. Shatin et al (1986) reported on the first use of this implantable neurostimulator in patients with chronic low back and leg pain.^49^ This early neurostimulator offered several programming modes, including automatically cycling therapy with timed ON and OFF settings. Interestingly, the 90 patients with follow-up data, averaging 14.5 months, reported better pain relief with stimulation in cycling mode (typical programming: 64 seconds on, 1 minute off) than with continuous stimulation. Another advantage was that the physician could more readily control patient access to the parameters and prevent the patient from arbitrarily modifying the therapy.^49^

Shifting to an implantable neurostimulator introduced additional technical considerations for patient safety, including a hermetically sealed casing, self-sealing connectors with captured set screws, insulation of the case to prevent muscle stimulation, and ensuring long-term biocompatibility.^49^ From the earliest use of implantable neurostimulators, it was recognized that patients would likely need additional medical procedures. As a result, early device designs accounted for safety during interventions such as electrocautery and defibrillation.^49^

### First patient programmer

The early RF-powered SCS systems, which required patients to wear an external power source to maintain therapy, allowed patients to make programming adjustments within physician-prescribed parameters, but the need for an external power source limited convenience and usability, particularly for individuals seeking a solution with an implantable battery. Despite these drawbacks, RF systems provided a foundational step in empowering patients to take an active role in managing their therapy.

A small patient programmer controlled the first implantable neurostimulator, allowing patients to make their own programming adjustments within parameters preset by their physician.^49^ This was particularly useful for patients far from the center where their SCS therapy was implanted and managed. In these first neurostimulators, reed switches were still incorporated to turn the device on and off using an external magnet, stemming from cardiac pacing, which allowed emergency rooms to turn off “runaway” pacemakers quickly.^24^ Eventually, neurostimulators abandoned reed-switch technology due to safety concerns, such as accidental deactivation caused by environmental magnets. This change allowed on-off control only by the patient or clinician programmer.^66^

The Medtronic EZ Patient Programmer (Figure 2F), used with the Itrel 3 device, was investigated in a 5-year clinical study published in 2003.^66^ The Itrel 3's on-off functionality was controlled either by a magnet or a programmer. The study observed patient satisfaction with the system; metrics included characterization of amplitude, pulse rate, and pulse width adjustment, as well as the ease and frequency of programmer use compared with the reed switch magnet. Almost all patients (95%) were given the ability to adjust amplitude. At 1-month postimplant, 55% of patients reported changing the stimulation amplitude daily, while 17% made no changes. Patients reported making fewer adjustments over the course of the study. By the 5-year follow-up, 50% of patients had not made changes since the last visit, and only 29% were making daily programming adjustments.^66^

Besides turning the device on/off, the patient programmer helped address the variability in stimulation sensation during positional changes. Optimal stimulation amplitudes in an upright seated position are typically higher than therapeutic amplitudes in a supine position.^66^ The patient programmer allowed the patient to make therapy adjustments needed for comfortable stimulation and pain relief.

Today’s patient programmers provide essential information about the SCS system, such as the battery status of a rechargeable device and whether the system is MR Conditional, which is critical for determining MRI eligibility. Ensuring the security of the interaction between the external programmer and the implant is equally essential, as it protects the system from unauthorized access. To address this, some systems incorporate a communicator tool that facilitates secure communication between the programmer and the implant. While the communicator enhances security and functionality, it introduces an additional external component that patients must manage, potentially increasing complexity and reducing therapy adherence.

### Battery technology

The characteristics of the battery within the neurostimulator will impact a patient’s experience with SCS therapy. As discussed above, a primary cell (or recharge-free battery) will typically limit programming parameters to avoid early battery depletion. Rechargeable batteries offer more programming options but require patient interaction for recharging the device. Research and development of battery technologies customized to the distinct needs of patients with SCS systems has been an essential cornerstone of SCS therapy innovation.^67^

Battery performance, impacted by the development of waveforms with high energy requirements, has become a critical factor for patient satisfaction with the SCS system. With long-term use and repeated recharging, traditional lithium-ion rechargeable batteries undergo capacity loss (ie, capacity fade).^68^ As battery capacity diminishes over time, patients must recharge more frequently because the battery retains less charge with each cycle.^69^ In addition, patients using higher-energy waveforms may need to recharge more often; faster recharge capabilities are ideal for patients, but for traditional lithium-ion batteries, hastening recharge can also cause battery degradation. For patients with rechargeable SCS systems, battery capacity and rechargeability affect the patient’s therapy experience. Improved recharge capabilities and a consistent recharge experience throughout the therapy may enhance the patient experience with the device.

Understanding that patients using higher-energy waveforms have unique power demands, a new battery chemistry–featuring proprietary lithium-ion technology called Overdrive™—was developed to meet these needs. This battery chemistry allows stable performance with minimal capacity fade over the device’s lifetime, regardless of use conditions. Testing of frequent recharge showed that, with over 3300 charge cycles (one cycle per day over 9 years), the battery retained over 95% of its initial capacity.^69^ Results of the battery testing suggest capacity fade and battery exhaustion are independent of therapy energy demands.^67^ Overdrive technology also considers the patient recharge experience, enabling faster charging with four different charging speeds to meet patient recharge and comfort needs.^70^ At the fastest rate, a device can be recharged from empty to full in about an hour, three times faster than traditional lithium-ion batteries.^71^ The slower recharging rates help mitigate recharge-induced heating, which can be uncomfortable for patients when recharging at faster rates. A recent survey showed that patients rated the overall charging convenience of an SCS system with Overdrive technology significantly higher and reported significantly fewer stimulation interruptions than those using non-Overdrive systems.^72^ Although fast recharging generates heat, testing has shown that this system is unlikely to cause any tissue injury.^73^

Ideally, enhancements in battery performance will improve the patient experience with rechargeable systems. Historically, patient frustration with recharge burden has been considered a significant contributing factor to the abandonment of SCS therapy, lack of SCS effectiveness, and early device explants. Van Buyten et al. (2017) reported a higher explant rate for conventional rechargeable SCS systems than for nonrechargeable ones. They speculated that the recharge burden may reduce patient compliance with the therapy.^74^ To this point, some cost-effectiveness studies have found nonrechargeable systems to be more cost-effective than rechargeable systems when assessing explants performed for any reason.^59^ Other analyses point to the cost-effectiveness of rechargeable systems.^56^^,^^58^ These findings highlight the critical role of battery performance and usability in long-term therapy success and device choice.

Improvements in battery technology may also impact patient comfort by enabling smaller device designs. A known issue patients may face is pain or discomfort at the neurostimulator site, which is distinct from the acute pain more commonly experienced shortly after implant surgery. These complications may require revisions or removal of the system, compromising the efficacy of the therapy. It is hypothesized that battery size and body type may contribute to pain or discomfort at the neurostimulator implant site. In one study of 764 patients, small rechargeable batteries (<30 cm^3^) were associated with a lower incidence of pocket site pain than large rechargeables (>30 cm^3^). However, nonrechargeable devices (>30 cm^3^) had the lowest occurrence of implant site pain (*P* = 0.048).^75^ An additional study examined pocket-site pain in patients implanted with SCS systems from various manufacturers; the neurostimulators were 48–55 mm in height, 46–54 mm in width, and 10.8–12.5 mm in depth.^76^ While these slight differences in size did not influence pocket site pain, the authors found that patients with smaller bodies, particularly women, were more likely to experience pocket pain. In addition, underlying causes of this pain included patient weight loss and contact with the waistbands of clothing. Advances in battery technology have enabled the development of increasingly smaller neurostimulators, enhancing patient comfort and improving the overall therapy experience. This evolution is evident when comparing early models, such as Medtronic’s Itrel II with a volume of 23 cm^3^, to the current smallest and thinnest model available, Inceptiv™ (Medtronic), which has a volume of 13.77 cm^3^.^77^ Modern SCS systems that use external RF or microwave power sources—such as those manufactured by Nalu and Curonix—offer the advantage of a minimal implant profile, avoid the need for surgical replacement due to battery depletion, and may be small enough to allow percutaneous implant. However, like earlier RF-based systems, they share the limitation that therapy is only delivered when the external power source is actively connected.

### Accelerometer technology and the first adaptive SCS system

**Figure 3:**
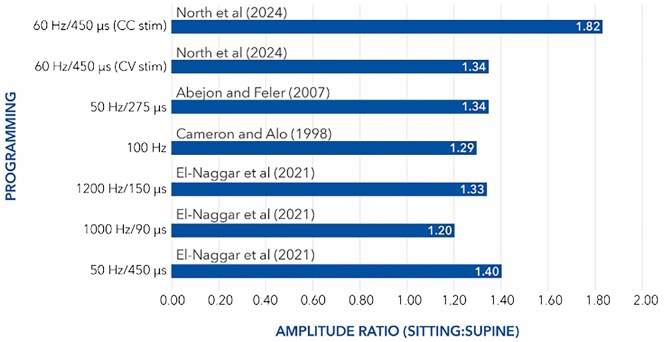
The amplitude required for therapy perception is consistently higher in an upright seated position than in a supine position, as demonstrated by the sitting-to-supine amplitude ratio reported across studies. This ratio is also consistent across different studies and stimulation parameters.^78–81^

Therapy adjustments are a significant challenge for patients who change positions, with increased amplitude needed when transitioning from upright to supine (Figure 3).^78^^,^^79^^,^^82^ Spinal cord movement, averaging 2.2 to 3.4 mm during position changes, is a major contributor to changes in stimulation sensation.^83^ Patient surveys indicated that stimulation becomes uncomfortable for over 70% of patients when they change positions, and they respond by making stimulation adjustments either before or after the posture change.^84^ Patients may also set the therapy to a suboptimal level to avoid position-related discomfort.

**Figure 4:**
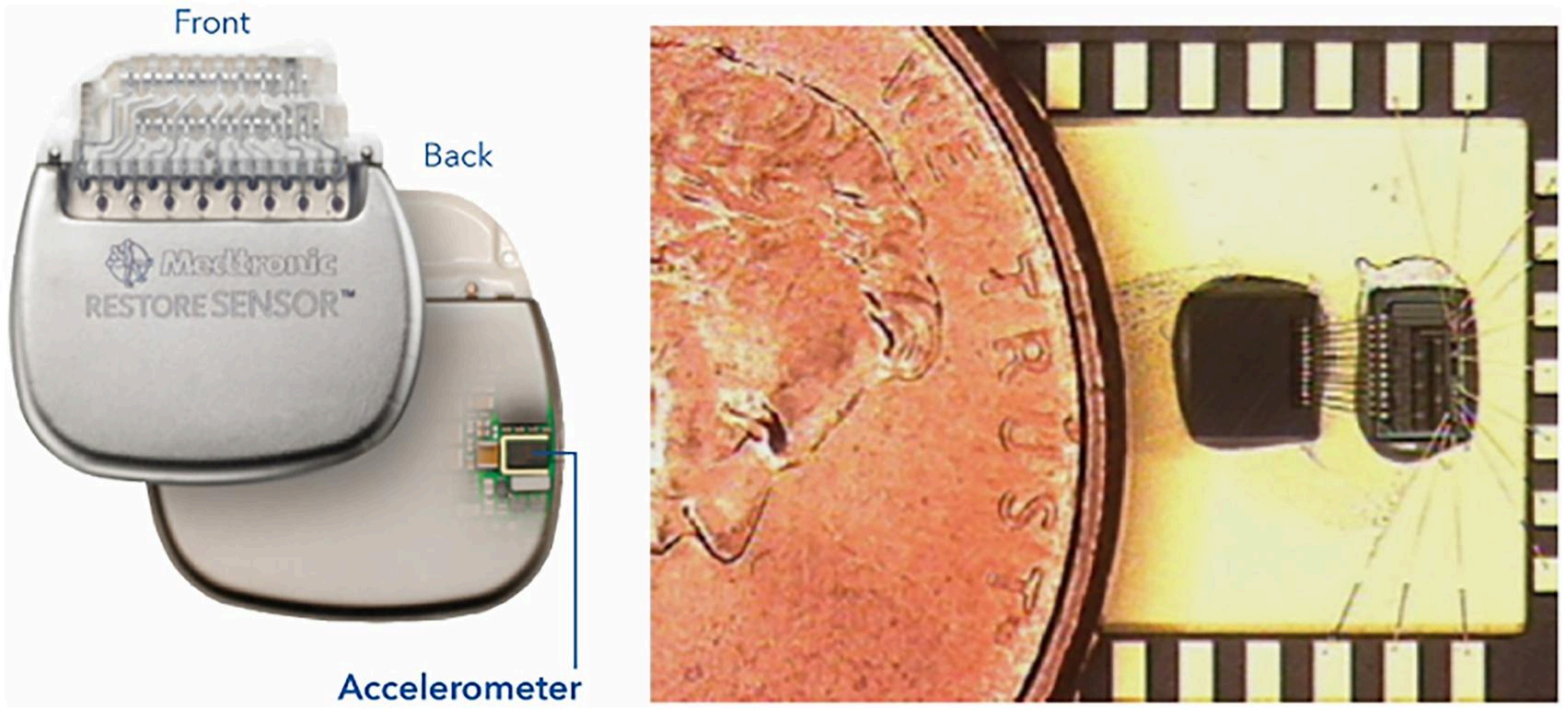
An accelerometer from the RestoreSensor™ device is shown relative to its location in a neurostimulator (left) and its size compared to a penny (right). This inertial sensor consists of a micro-electrical-mechanical sensor and an integrated circuit. The final sensor is encapsulated for manufacturing. Reprinted with permission from © Medtronic, Inc. —all rights reserved.

Initially, patient programmers allowed users to adjust for position-related stimulation changes manually, but this placed the burden on the patient. A more innovative technical solution involved embedding a 3-axis accelerometer within the neurostimulator (Figure 4) to detect trunk orientation by measuring deflections of internal weights in response to gravity. Adapted from commercial accelerometers used in mobile technology and gaming, this innovation enabled the neurostimulator to track posture in 3 dimensions (left vs right; up vs down, back vs front). By assigning preset stimulation amplitudes for seven positions–upright, mobile, reclining, lying left, lying right, lying front, and lying back—the system could automatically adapt stimulation based on objective feedback. This position-adaptive stimulation marked the first use of sensing technology to deliver patient-preferred stimulation programs in response to common body positions.

The feasibility of this technology was first demonstrated in a prospective, multicenter, open-label, randomized feasibility trial involving 20 patients, in which automatic adjustments via an external accelerometer were compared with manual adjustments.^85^ The results supported the feasibility of automatically adjusting stimulation and showed that patients preferred automatic stimulation due to greater convenience and satisfaction. Subsequent studies, including the FDA-mandated Restore Sensor Study, confirmed these findings.^8^ Among 76 subjects, 86.5% reported better pain relief without loss of convenience, or better convenience without loss of pain relief. Overall, 88.7% of participants reported greater pain relief with position-adaptive stimulation than with conventional stimulation. In addition, 87.3% and 90.1% of subjects preferred and intended to continue to use AdaptiveStim technology, respectively.^8^

Beyond position-adaptive stimulation, accelerometer integration enables tracking of patient position patterns, providing valuable data for physicians to assess function and initiate discussions on activity levels.^86^ Wearable sensor technology is increasingly used in pain medicine to monitor and enhance patient outcomes, underscoring its relevance in SCS therapy.^87^ Wearable tools are employed to track daily pain levels, predict and prevent pain flare-ups, improve home rehabilitation, and/or improve the delivery of pain care.^88^^,^^89^ Sensor and wearable technologies are increasingly being incorporated into the monitoring of SCS therapy.^90^^,^^91^

### Neurophysiological sensing capabilities

While position-adaptive stimulation automatically adjusted for significant position changes, it did not address spinal cord movements unrelated to position. Patients continued to experience overstimulation during activities such as laughing, coughing, stretching, and other activities not differentiated by the accelerometer (sitting, standing, walking, biking).^92^ Dynamic spinal cord movement and changes in the distance between the lead and the spinal cord target continue to underlie inconsistent SCS therapy doses.

An elegant and practical solution to the issue of inconsistent dose due to cord movement is to measure the evoked compound action potential (ECAP) generated by the SCS pulse to obtain insight into the volume of tissue being activated per pulse. The ECAP signal can be used as an input to control the level of stimulation delivered. With the ultimate goal of maintaining a consistent ECAP signal, the result is a consistent and comfortable therapy experience for the patient. From a practical perspective, the ECAP is a logical signal to measure since it corresponds closely to the perception level of the patient.^93^ From a technical standpoint, the methods for detecting and analyzing the ECAP can impact the reliability of the feedback loop and its suitability for different therapies.^94^ Filtering and detecting ECAPs near the perception threshold enable algorithms tuned for subthreshold programming, preventing therapy amplitude from exceeding the clinician-prescribed level.

The foundation for developing ECAP-based closed-loop SCS systems was established through recordings from patients undergoing SCS for pain relief.^95^^,^^96^ Computational modeling has aided in the characterization of ECAPs, showing that axons with diameters of 8.7 to 10.0 µm located in the dorsal aspect of the spinal cord are the dominant contributors to the ECAP, and that electrode position relative to the spinal cord, lead lateral shift, and thickness of the dorsal cerebrospinal fluid layer on ECAP amplitude also impact the ECAP.^97^ Modeling has also demonstrated the importance of cord position and pulse width on the resulting ECAP.^98^

Saluda Medical was the first company to commercialize a closed-loop SCS system, the Evoke™ neurostimulator, employing ECAPs. This system was limited to traditional, low-frequency SCS waveform stimulation.^11^ The classical application of closed-loop control at low frequencies enables the system to dynamically adjust the output to maintain a desired ECAP size, aiming to deliver a consistent, optimal stimulation dose while avoiding over- and under-stimulation.

The double-blind EVOKE RCT demonstrated the clinical advantages of ECAP-based closed-loop (CL) SCS over conventional open-loop (OL) or fixed-output SCS. In this study, 134 subjects were randomized to receive either CL-SCS or OL-SCS, both programmed to low-frequency stimulation (mean ∼40 Hz; range 10–80 Hz).^11^ At the 2-year follow-up, the responder rate for overall pain was significantly higher in the CL-SCS group (79.1%) compared to the OL-SCS group (53.7%).^99^ After the 2-year follow-up, 40 subjects crossed over from OL to CL, and 80% chose to remain on CL-SCS. At 3 years, the responder rate remained higher in the CL-SCS group (77.6%) vs the OL-SCS group (49.3%).^100^

In addition to supporting closed-loop with low-frequency therapy similar to the Saluda system, the Medtronic Inceptiv™ SCS system was designed to expand closed-loop capabilities for conventional and contemporary waveforms. This broadens the application of closed-loop SCS to include high-frequency stimulation delivered at amplitudes near the perception threshold—extending its use beyond conventional paresthesia-based approaches.^101^ The primary goal of closed-loop programming when used with DTM SCS, typically set at a lower amplitude, is to prevent overstimulation events and ensure stable, consistent therapy.^102^ Patients using closed-loop SCS with DTM SCS and other programming options have been studied in a single-arm prospective study reporting an 86% responder rate for overall back and leg pain at 3 months (*n* = 51).^103^

While the potential of ECAPs-based CL control to enhance pain relief is promising, only the EVOKE RCT has demonstrated improved pain outcomes—and only with low-frequency conventional stimulation. For modern waveforms, such as DTM™ SCS, whether CL control adds further benefit remains unknown. Emerging evidence from a prospective study, in which 72% of subjects were programmed to CL with DTM™ SCS, suggests that CL-technology may offer advantages for modern therapies in addition to low frequency. Subjects in the study were able to use therapy near their pain perception threshold, comfortably engage in activities of daily living, and achieve meaningful pain relief, with 86% reporting at least a 50% reduction in pain.^103^ While these findings are encouraging, they are preliminary, and further well-controlled studies are needed to determine whether CL control provides a significant incremental benefit when paired with DTM™ SCS.

### Full-body MR conditional systems

The ability to undergo an MRI is an essential consideration for patients with active implanted medical devices (AIMD), such as a neurostimulator. Hazards of AIMDs in an MRI environment include magnetic field interactions, force exerted on the stimulator, induced stimulation, and device damage and heating.^104^ Heating occurs when the implanted lead acts like an antenna, amplifies the RF field, and causes local increases in temperature; heating is a particular concern due to the potential for tissue damage.^104^

While computed tomography (CT) scanning can be an acceptable diagnostic imaging tool for some patients, MRI is the preferred imaging modality in many disease states, including spinal disorders, large joint disorders, stroke, multiple sclerosis, and malignancy.^105^^,^^106^ Up to 84% of SCS patients are expected to need an MRI within 5 years.^107^ Within 10 years of SCS implant, 98% of SCS patients are expected to need an MRI, and 59%–74% will require a non-spine MRI.^107^ A related claims analysis found that up to 74% of SCS patients will need a scan specifically of the trunk (ie, chest, spine, abdomen, pelvis, heart, or breast) within 5 years of their SCS implant; this rate increases to 87% by 10 years postimplant.^108^ In addition to MRI scans related to the patient’s main pain condition, 87.1% of chronic pain patients have at least one other comorbid condition that may require an MRI.^109^ These data confirm that SCS patients will eventually need an MRI, and that the scan will most likely be ordered by a physician who is not treating their pain condition and may not be aware of the scanning conditions for SCS systems.

Access to MRI scans clearly affects patient care. Still, MR Unsafe SCS systems or MR Conditional requirements can limit eligibility, forcing reliance on alternative imaging modalities that may not be ideal. In some cases, MR Unsafe SCS systems must be explanted, requiring unnecessary surgical procedures and removal of effective pain therapy. Even with MR Conditional SCS systems, patient care may be delayed as physicians, radiologists, and company representatives ensure all conditions are met. If the conditions are not met, options include an alternative scan or explantation of the system.

**Figure 5:**
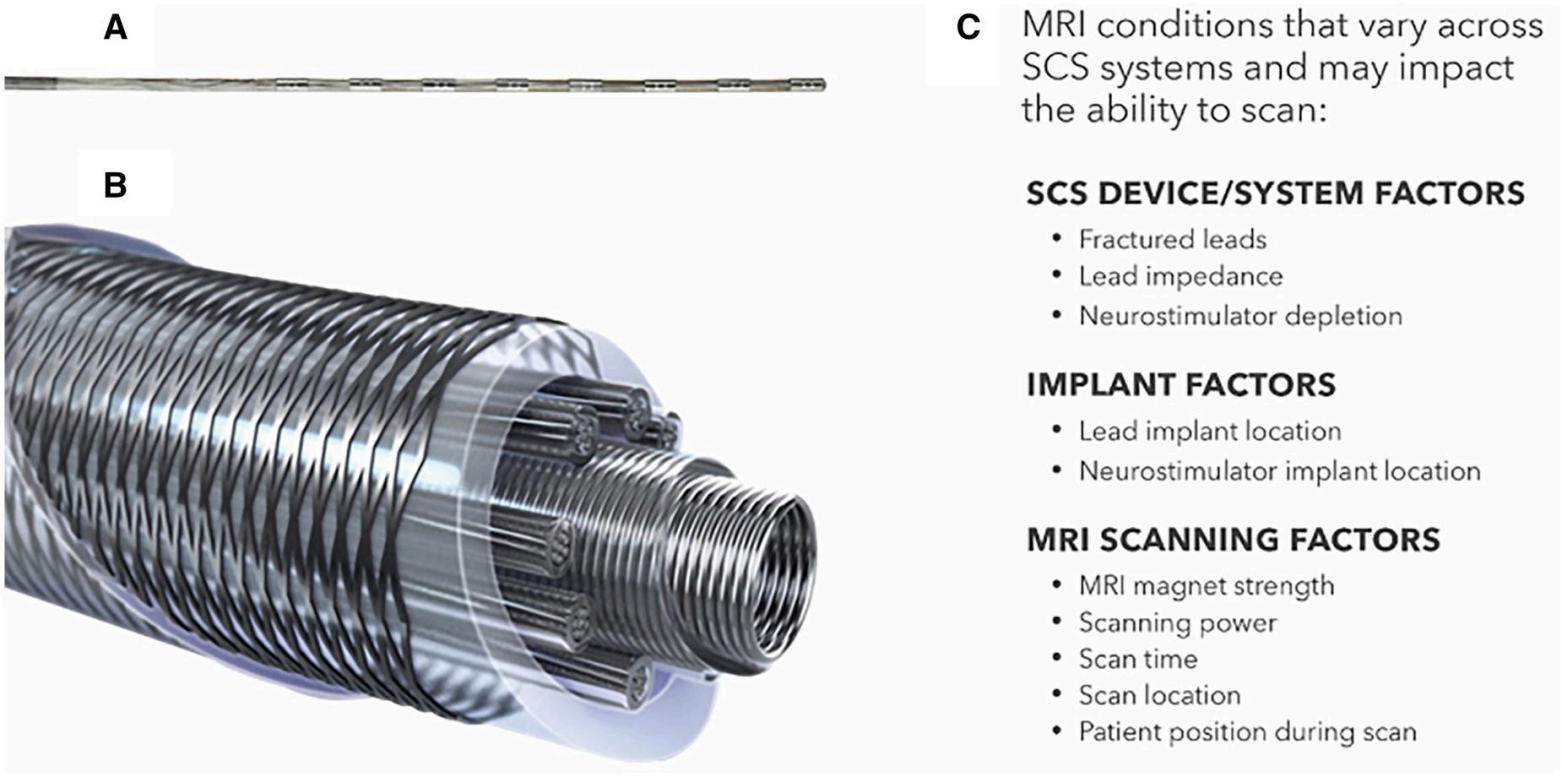
(A) Medtronic percutaneous lead with SureScan™ MRI technology. (B) Medtronic’s tantalum RF shielding reduces RF electrode heating, which is the most significant MRI-related risk to SCS patients. Moreover, the tantalum shielding makes the RF electrode heating characteristics of the lead insensitive to the presence of shorted or fractured lead wires, and therefore, no lead impedance measurement is required to establish MR eligibility. (C) While many SCS systems are MR Conditional, the conditions under which SCS systems can be scanned vary from system to system. Understanding and appreciating the factors that may limit patients’ access to MRI scans is essential. Reprinted with permission from © Medtronic, Inc. —all rights reserved.

Advancements in MR Conditional devices began with cardiac implants, such as Medtronic’s Revo MRI pacemaker (released in Europe in 2008; FDA approved in 2011), and have since extended to neuromodulation systems.^110^ A second-generation device, the Advisa MRI, was introduced in the United States in 2013.^111^ This foundation and expertise with cardiac devices led the way for MR Conditional SCS devices. Early SCS systems allowed limited MR Conditional labeling for head scans, but full-body MRI access became a recognized need.^112^ This was an important step, but only about 25% of MRI scans obtained in the United States are brain scans.^113^ Two strategies enabled full-body SCS MRI scans: Testing safe scanning conditions without hardware changes or developing new lead designs and neurostimulator technology. Medtronic addressed RF heating risks, the primary concerns for SCS systems in MR environments, by developing novel tantalum shielded leads (Figure 5A) and feedthrough filter capacitors to minimize RF energy accumulation and prevent tissue injury due to RF heating (Figure 5B).

Furthermore, the tantalum shielding protects the patient from injury at fracture sites, where heat can accumulate.^71^ Extensive safety testing, including 100 million simulated scans performed with 10 000 patient scenarios, led to the 2013 commercial release of SureScan full-body MR Conditional systems in the United States (Table 1).

Today, all major SCS manufacturers, including those with implanted neurostimulators or systems powered by battery-free RF or high-frequency electromagnetic-coupled technology, offer full-body MR Conditional systems. However, conditions differ between manufacturers and systems (Figure 5C), with some labeling significantly limiting access to standard clinical MRI protocols by requiring reduced RF power or limited scan duration. SCS systems without RF-shielded leads require a lead impedance check to establish MR eligibility. High impedance or lead fractures—common clinical occurrences—can render otherwise MR Conditional systems unsuitable for MRI. A retrospective study revealed that 18.5% of non-SureScan leads across manufacturers exhibited at least one high-impedance contact after an average follow-up period of 2.25 years, rising to 43% by the fifth year post-implant.^114^ This loss of MRI access can have profound consequences, as detailed by Jotwani et al (2021).^115^ The first patient experienced a likely stroke after a transcatheter aortic valve replacement, but could not undergo an MRI scan due to high impedance. The medical team considered immediate explantation of the SCS system, but the associated surgical risks outweighed the potential benefits.^115^ A second patient, with a history of radiculopathy and post-back surgery pain, presented with new back pain and suspected lumbar disc herniations. Instead of an MRI scan, a CT scan was performed when the system could not be placed into MRI mode.^115^ The SCS system was eventually explanted due to the need for multiple MRI scans and the SCS device’s failure to meet the required conditions. The final patient with metastatic melanoma lost access to MRI surveillance due to high impedance in the SCS system.^115^ As mentioned in one of the cases, CTs may be attempted instead of an MRI and may be an acceptable alternative for some patients.

Unfortunately, the confusion and complexity around MR Conditional labeling for SCS devices have led some imaging centers to refuse to scan patients with SCS devices. Moreover, patients and physicians should understand that specific conditions must be met for a safe MRI scan and that device-related conditions, such as impedance, may change over time. The potential to lose MRI conditionality due to high impedance is essential for the physician and patient to consider. Finally, not all SCS systems have the same MRI conditionality. A robust development and testing strategy has ensured patients with Medtronic MR Conditional SCS systems have uncomplicated access to MRI scans.

### Limitations

While technological advancements across the industry have enhanced the SCS therapy experience for individuals living with chronic pain, opportunities to further support patients and optimize outcomes remain. Beyond new technology, setting appropriate expectations for SCS therapy is essential. A survey of patients undergoing SCS for postsurgical back pain revealed a limited understanding of both pain and the therapy itself.^116^ Patients identified a gap in practical information, expressing a need for timely, actionable guidance—such as clear “do’s and don’ts” related to therapy use. Other concerns included the impact of recharging and device placement on daily life.^116^

Similarly, another patient-centered survey highlighted challenges with the neurostimulator’s physical placement. Participants reported difficulty wearing certain clothing, discomfort during specific movements, and dissatisfaction with body image.^117^ Some also experienced trouble connecting to the recharger and noted that recharging had to be deliberately incorporated into their daily routines.^117^ Structured interviews with individuals using SCS therapy continue to shed light on these and other therapy-related challenges.^118^

As technology evolves, healthcare systems, manufacturers, and stakeholders must work collaboratively to ensure that innovations are accessible, usable, and effectively integrated into clinical practice. For example, the emergence of remote programming has introduced new considerations, including the need for reliable Wi-Fi, familiarity with digital tools among providers, cybersecurity concerns, and complex reimbursement systems.^61^ These factors underscore the multifaceted nature of SCS therapy—advancements do not occur in isolation and must be supported by education, infrastructure, and policy.

## Conclusions

Since the first clinical case, the landscape of SCS therapy has evolved significantly. Over the past decades, collaborative efforts and research spearheaded by industry manufacturers and academic investigators have driven continued enhancements and refinements of this therapy for patients with chronic pain. Key SCS technology innovations include patient programmers, enhanced rechargeable battery technology, position-adaptive therapy, full-body MRI conditionality, integration of innovative waveforms, and sensing-based closed-loop features. While these advancements are significant, ongoing innovation remains essential to optimize therapy outcomes further and address the evolving needs of patients with chronic pain.

## Disclosures

This supplement was sponsored by Medtronic, which provided funding for its publication. No authors received compensation for their contributions to the writing, review, or critical input into the content of this article. J.G. and L.J. are Medtronic employees and participated in the manuscript writing and editing. All authors critically reviewed the manuscript and approved the final version.

BOX 1 CURRENT TECHNOLOGICAL ADVANCEMENTS IN SCS: PERSONALIZATION AND OPTIMIZATION OF THERAPY

Present-day innovations in spinal cord stimulation therapy are driven by advances in remote programming, wireless data transfer, and big data analysis using artificial intelligence (AI). These technological tools are ushering in a new era of personalized and efficient patient care. Remote programming has allowed healthcare professionals to fine-tune and adjust SCS parameters without in-person visits, increasing patient convenience and reducing healthcare costs.^61^^,^^62^ Bluetooth-enabled data transfer streamlines communication between the implanted device and external devices, supporting real-time collection of data such as programming, stimulation usage, battery usage, lead status, and patient physiology.^61^ These capabilities are especially important for closed-loop SCS systems, which rely on efficient data transfer for features like ECAP feedback. Mobile digital health platforms further support patient education and enable real-world data collection for better characterization of the patient experience.^63^^,^^64^ Machine learning algorithms can now analyze vast data sets to predict long-term response to SCS, helping to personalize therapy efficacy and reduce trial-and-error approaches.^65^ Collectively, these technologies contribute to a more responsive therapy, fostering improved outcomes and a higher quality of life for patients with chronic neuropathic pain.
